# The efficacy and safety of Yuxingcao eye drops in the treatment of COVID-19 conjunctivitis: A protocol for a systematic review

**DOI:** 10.1097/MD.0000000000023093

**Published:** 2020-12-04

**Authors:** Jiajun Wu, Liqu Pu, Hui Zhou, Wenjia Qu, Dandan Zhao, Chunmeng Liu, Xuewen Dong, Fuwen Zhang

**Affiliations:** a School of Eye; b School of Nursing; c Department of Endocrinology, Chengdu University of Traditional Chinese Medicine, Sichuan Province, P.R. China.

**Keywords:** COVID-19 conjunctivitis, protocol, systematic review and meta-analysis, Yuxingcao eye drops

## Abstract

**Background:**

Coronavirus disease 2019 (COVID-19) is a global pandemic caused by the Severe Acute Respiratory Syndrome Coronavirus-2 (SARS-CoV-2). There is no specific cure for this disease, and the clinical management mainly depends on supportive treatment. This disease may affect SARS-CoV-2 conjunctivitis. Yuxingcao eye drops is used in treating COVID-19 conjunctivitis in China.

**Methods:**

A comprehensive literature search will be conducted. Two methodological trained researchers will read the title, abstract, and full texts and independently select the qualified literature according to inclusion and exclusion criteria. After assessment of the risk of bias and data extraction, we will conduct meta-analyses for outcomes related to COVID-19 conjunctivitis. The heterogeneity of data will be investigated by Cochrane X^2^ and *I*^2^ tests. Then publication bias assessment will be conducted by funnel plot analysis and Egger test.

**Results:**

The results of our research will be published in a peer-reviewed journal.

**Conclusion:**

Our study aims to systematically present the clinical evidence of Yuxingcao eye drops in treating COVID-19 conjunctivitis, which will be of significant meaning for further research and clinical practice.

**PROSPERO registration number:**

PROSPERO CRD42020209059.

## 1 Introduction

Coronavirus disease 2019 (COVID-19) is a global pandemic caused by the Severe Acute Respiratory Syndrome Coronavirus-2 (SARS-CoV-2).^[1,2]^ The patients of COVID-19 usually present with fever, cough, while about 23.7% of patients are accompanied by at least one coexisting disease.^[3–7]^ A study described the presence of viral antigens on the conjunctiva surface, as well as detection in conjunctival swabs suggesting that ocular secretions are potentially infectious.^[8]^

The ocular findings of coronavirus infection in humans are not clear. Human coronavirus infection was first reported in the Netherlands (HCoV-NL63) as a cause of respiratory infection.^[9]^ In subsequent studies of coronavirus infections, the clinical manifestation of eye involvement was conjunctivitis in 17% of the cases, presenting primarily in children.^[10]^ In cases of human coronavirus infection SARS, HCOV-NL63, and SARS-CoV-2 in adults involvement of the anterior segment of the eye, such as conjunctivitis, they have been postulated an infection route in the conjunctiva with the virus migrating in the lacrimal fluid through the duct inferior meatus until the respiratory epithelium to start its replication.^[11,12]^ It is noteworthy that the ocular mucosa and the respiratory epithelium share many characteristics such as the receptor alpha 2–3 linked to sialic acid, it found in the tract lower respiratory and ocular tissue mainly in epithelial cells of the conjunctiva, could be related to the tropism of many viruses such as Influenza virus.^[13]^ In China, traditional Chinese medicine (TCM) is widely used in treating COVID-19.^[14,15]^ Among the many commonly used TCMs, the most commonly used one is Yuxingcao eye drops.

## 2 Methods and analysis

### 2.1 Study registration

This systematic review protocol was registered with PROSPERO 2020 (registration number: CRD42020209059). And the protocol report is in the base of the Preferred Reporting Items for Systematic Reviews and Meta-Analyses Protocols (PRISMA-P) declaration guidelines.^[16]^ The review will be performed in line with the PRISMA declaration guidelines.^[17]^

### 2.2 Inclusion and exclusion criteria

#### 2.2.1 Study design

In this study, both randomized studies and non-randomized studies will be included. Randomized studies can provide reliable clinical evidence, but it can be time consuming and expensive. Non-randomize studies may lead to greater bias, but it is more convenient to obtain clinical data. Since COVID-19 is an urgent public health event, it is difficult to carry out randomized studies, it is appropriate to include nonrandomized studies in this systematic review and meta-analysis.

#### 2.2.2 Participants

Participants with laboratory-confirmed COVID-19 conjunctivitis will be included in this study. The assay was primarily RC-qPCR, and there were no restrictions on the age, sex, or disease severity of the participants.

#### 2.2.3 Interventions

Studies using Yuxingcao eye drops will be included. There will be no restriction about the doses and methods of use of intervention. Also, there will be no limitation about control group.

#### 2.2.4 Outcomes

Since there are no core outcome sets for COVID-19, it is difficult to predefined what outcomes will be included in our study. In general, any outcome that can reflect the condition will be included in this study.

### 2.3 Study search

Three English database including PubMed, EMBASE, Cochrane Library Central Register of Controlled Trials, and 4 Chinese databases including China National Knowledge Infrastructure (CNKI) database, Wanfang Data Knowledge Service Platform, the VIP information resource integration service platform, China Biology Medicine Disc will be searched from its inception to September 10, 2020. There will be no language limitation. Preprinted website including arXiv (http://arxiv.org/), BioRxiv (https://www.biorxiv.org/), F1000 (https://f1000.com/), and PeerJ Preprints (https://peerj.com/preprints/) will also be searched to find out more unpublished manuscript. Chinese Clinical Trial Registry (ChiCTR) and ClinicalTrials.gov will also be searched to find out ongoing research. The references of included manuscript will be searched.

A search strategy of the combination of controlled vocabulary and text words will be adopted. Boolean operators will be used to concatenate search terms. This work will be conducted by 2 authors (JW) independently. The search strategy of PubMed is presented in Table 1.

**Table 1:** Cochrane Library search strategy.

Number	Search terms
1	Mesh descriptor: (Yuxingcao eye drops) explode all trees
2	((((((Yuxingcao eye drops[Title/Abstract]) OR Eye drops[Title/Abstract]) OR Yuxingcao [Title/Abstract]) OR Traditional Chinese medicine eye drops[Title/ Abstract]) OR Traditional Chinese medicine[Title/Abstract])
3	Or 1–2
4	Mesh descriptor: (COVID-19 conjunctivitis) explode all trees
5	((((((2019 novel coronavirus infection[Title/Abstract] OR 2019-nCoV infection[Title/Abstract]) OR COVID-19 pandemic[Title/Abstract]) OR coronavirus disease-19[Title/Abstract]) OR 2019-nCoV disease[Title/Abstract]) OR COVID19 conjunctivitis[Title/Abstract]) OR 2019 novel coronavirus disease[Title/Abstract]) OR conjunctivitis[Title/Abstract]
6	Or 4–5
7	3 and 6

Example of PubMed search strategy.

### 2.4 Study selection

**Figure 1:**
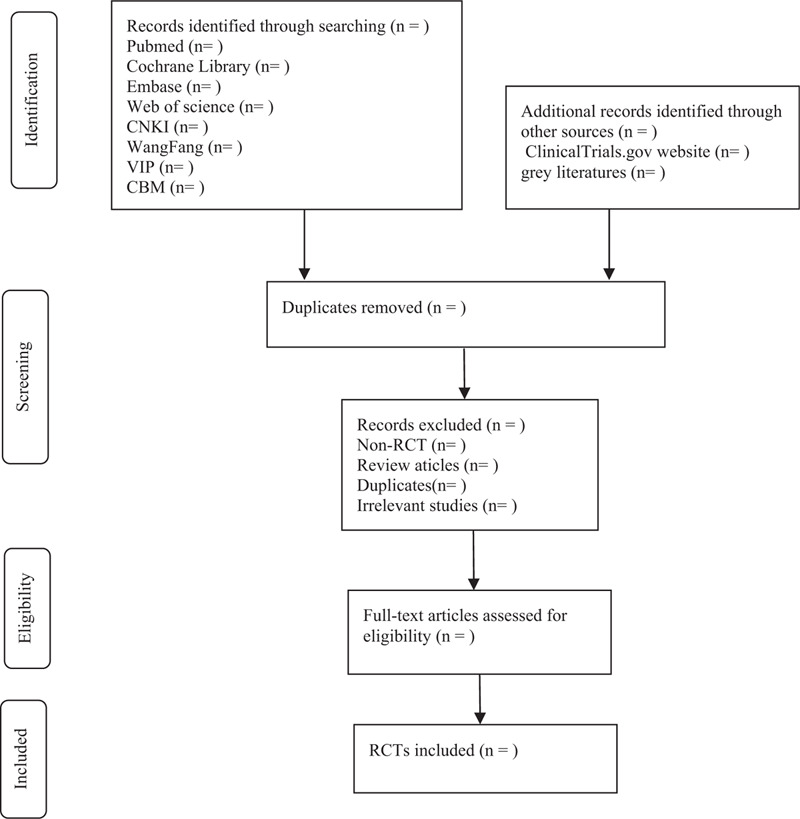
Flow chart of the study selection.

EndNote X9 will be used by 2 researchers (JW and LP) to screen the citations independently according to the predefined inclusion and exclusion criteria. Discrepancies between 2 authors will be solved by discussion with a third author (HZ). A research flow chart will be drawn to show the whole process of research selection (Fig. 1).

### 2.5 Data extraction

Data extraction will be conducted by 2 independent authors (JW and LP) according to a prespecified form and checked by a third author (FZ). The following data will be extracted: the first author's name, publication time, country, article title, article type, interventions in experimental and control group, course of treatment, severity of disease, number of patients in each group, ages and sex of patients, outcomes and adverse effect. If the author does not report certain information in the article, we will then contact the authors by email for more detailed information. Once the extraction is complete, the 2 authors will check with each other to ensure the accuracy of the data.

### 2.6 Risk of bias assessment

Different risk of bias assessment tools will be used according to different types of research. The risk of bias of RCTs will be conducted using version 2 of the Cochrane risk-of-bias tool for randomized trials (RoB2).^[18]^ The Risk of Bias In Non-randomized Studies of Interventions (ROBINS-I) tool will be used to assess the risk of bias of non-RCTs according to Cochrane Handbook.^[19]^

### 2.7 Data analysis

Data analysis will be conducted using Stata 14.0, StataCorp, TX. The effect measure of binary variable will be expressed as risk ratio (RR) or odds ratio (OR) and 95% confidence interval (CI). For continuous variables, 95% CI and mean difference (MD) or standardized mean difference (SMD) will be used. The number needed to treat (NNT) will be calculated for the interpretation of results. Cochrane X^2^ and *I*^2^ tests will be conducted to assess the heterogeneity analysis between studies. When *P* < 50% and *I*^2^ > 50%, a random effect model will be used. When *P* > 50% and *I*^2^ < 50%, then a fixed effect model will be used to calculate the effect size. The results of RCTs and non-RCTs will be analyzed and presented independently. Subgroup analysis will be conducted to explore the subgroup effects and investigate the source of heterogeneity. If there is a substantial heterogeneity and quantitative synthesis is not appropriate, the results will be presented in the form of tables and figures. Publication bias and small-study effects will be evaluated by funnel plot and statistically investigated by Egger test with a *P* value boundary of 50%.^[20]^

### 2.8 Ethics

This paper is a systematic evaluation and does not need to pass ethical review.

## 3 Discussion

The aim of this study was to summarize the efficacy of Yuxingcao eye drops on COVID-19 conjunctivitis to provide an accurate guide for further research and clinical application. This study has some highlights. First, in order to collect clinical evidence as comprehensively as possible, preprinted websites will be searched in addition to main databases for systematic review. In addition, given the speed at which the epidemic is developing and the difficulty of conducting clinical trials, we will include both randomized studies and nonrandomized studies in the research. Nonrandomized studies have more biases and confounding than randomized studies, so the results of the 2 types of studies will be presented separately. In conducting the risk of bias assessment, we will use the latest version of the tool recommended in the handbook, which will methodologically ensure the correctness of our study.

## Author contributions

**Conceptualization:** Jiajun Wu, Liqu Pu.

**Data curation:** Hui Zhou, Dandan Zhao.

**Formal analysis:** Jiajun Wu, Liqu Pu.

**Funding acquisition:** Fuwen Zhang.

**Methodology:** Jiajun Wu, Wenjia Qu.

**Project administration:** Fuwen Zhang, Jiajun Wu.

**Resources:** Xuewen Dong, Chunmeng Liu, Fuwen Zhang.

**Software:** Jiajun Wu, Wenjia Qu, Fuwen Zhang.

**Supervision:** Fuwen Zhang, Jiajun Wu.

**Writing – original draft:** Jiajun Wu.

**Writing – review & editing:** Fuwen Zhang.
